# Factors Associated with Domain-Specific Asthma-Related Quality of Life in Adults with Asthma

**DOI:** 10.3390/healthcare14162626

**Published:** 2026-08-19

**Authors:** Emanuel Ionuț Poplicean, Alexandru Florian Crișan, Emanuela Tudorache, Bianca Mădălina Huidu, Daniel Alexandru Jipa, Roxana Mladin, Ion Costel Epuraș, Carina Gib, Cristian Oancea

**Affiliations:** 1 Doctoral School, “Victor Babes” University of Medicine and Pharmacy Timisoara, Eftimie Murgu Square 2, 300041 Timisoara, Romania; emanuel.poplicean@umft.ro (E.I.P.); bianca.huidu@umft.ro (B.M.H.); daniel.jipa@umft.ro (D.A.J.); roxana.mladin@umft.ro (R.M.); ion.epuras@umft.ro (I.C.E.); carina.gib@umft.ro (C.G.); 2 Center of Research and Innovation in Personalized Medicine of Respiratory Disease (CRIPMRD), “Victor Babes” University of Medicine and Pharmacy Timisoara, Eftimie Murgu Square 2, 300041 Timisoara, Romania; emanuela.tudorache@umft.ro (E.T.); oancea@umft.ro (C.O.); 3 Pulmonary Rehabilitation Center, Clinical Hospital of Infectious Diseases and Pulmonology, “Victor Babes”, Gheorghe Adam Street 13, 300310 Timisoara, Romania; 4 Research Center for the Assessment of Human Motion, Functionality and Disability (CEMFD), “Victor Babes” University of Medicine and Pharmacy Timisoara, Eftimie Murgu Square 2, 300041 Timisoara, Romania; 5 Pulmonology University Clinic, “Victor Babes” University of Medicine and Pharmacy Timisoara, Eftimie Murgu Square 2, 300041 Timisoara, Romania; 6 Centre for Translational Research and Systems Medicine, “Victor Babes” University of Medicine and Pharmacy Timisoara, Eftimie Murgu Square 2, 300041 Timisoara, Romania; 7 Oftalmo Sensory-Tumor Research Center-ORL (EYE-ENT), “Victor Babes” University of Medicine and Pharmacy Timisoara, Eftimie Murgu Square 2, 300041 Timisoara, Romania; 8 Center of Advanced Research in Cardiology and Hemostasology, “Victor Babes” University of Medicine and Pharmacy Timisoara, Eftimie Murgu Square 2, 300041 Timisoara, Romania

**Keywords:** asthma control, quality of life, treatment adherence, AQLQ

## Abstract

**Highlights:**

**What are the main findings?**

- Asthma control had the largest absolute standardized coefficient within each of the four domain-specific quality-of-life models.
- The inverse association between inhaler adherence and emotional functioning emerged only after adjustment for asthma control and was absent from the bivariate and ACT-excluded analyses, indicating a possible suppression effect.

**What are the implications of the main findings?**

- Assessing individual AQLQ domains provides more clinically relevant information than relying solely on the overall quality-of-life score.
- Asthma management should combine optimal disease control with targeted interventions addressing the specific factors influencing different quality-of-life domains.

**Abstract:**

**Background/Objective**: Asthma-related quality of life is related to multiple clinical, physiological, and behavioral factors. Nonetheless, the domain-specific associations of asthma control, inhaler adherence, lung function, and demographic characteristics remain poorly understood. This study aimed to examine the adjusted associations of asthma control, inhaler adherence, lung function, and age with individual domains of asthma-related quality of life. **Methods**: This cross-sectional observational study included 92 adults with asthma who demonstrated correct inhaler technique and adequate knowledge of their prescribed inhaler regimen. Asthma control was measured using the Asthma Control Test (ACT), inhaler adherence using the Test of Adherence to Inhalers (TAI), and quality of life using the Asthma Quality of Life Questionnaire (AQLQ). Pulmonary function was determined through spirometry. Spearman correlation analyses were initially conducted to identify associations between variables. Subsequently, separate multivariable linear regression models were created for each AQLQ domain, including ACT score, TAI score, age, and FEV_1_ % predicted as predictors. **Results**: A total of 92 adult patients with asthma were studied. Asthma control was significantly positively correlated with all AQLQ domains (all *p* < 0.001). In multivariable analyses, ACT had the largest absolute standardized coefficient within each of the four domain-specific models (standardized β range: 0.339–0.791). After adjustment, age was associated with the activity limitation and symptom domains, whereas FEV_1_ % predicted was associated only with the environmental stimuli domain. Inhaler adherence showed an inverse association with emotional functioning in the fully adjusted model (B = −0.094, 95% CI −0.158–−0.031; *p* = 0.0038). However, this association was absent in the bivariate analysis and in a sensitivity model excluding ACT (B = −0.015, 95% CI −0.067–0.037; *p* = 0.562), suggesting a possible suppression effect. **Conclusions**: ACT showed the largest standardized association within each domain-specific model, although the magnitude of its associations with the symptom and activity-limitation domains may partly reflect conceptual overlap between the instruments. The inverse TAI–emotional-functioning coefficient was model-dependent, did not meet the conservative multiplicity benchmark, and should be considered exploratory and hypothesis-generating. Additionally, lung function and age showed domain-specific associations, highlighting the need for a comprehensive, multidimensional approach in asthma management.

## 1. Introduction

Asthma is a chronic inflammatory condition of the airways, with a globally variable prevalence that, depending on the country, ranges from 1% to 29% of the population; it affects all age groups and is responsible for approximately 1000 deaths each day [1,2,3]. Despite advances in diagnosis, pharmacological treatment, and disease management, asthma continues to impose a substantial burden on healthcare systems and society, including reduced workplace productivity among adults and disruption of family life in pediatric populations [1,3,4,5,6]. AsmaCost, a study conducted in Spain, estimates that patients with poor disease control account for 70% of the total estimated global cost of asthma [7]. According to the Global Initiative for Asthma (GINA), optimal disease control relies primarily on the regular use of long-term inhaled medication, particularly inhaled corticosteroids, which reduce inflammation and the risk of exacerbations [1].

Disease severity is an important clinical factor associated with quality of life in patients with bronchial asthma [5,8], and the main therapeutic goal is to achieve disease control by identifying and addressing aggravating factors [7,9,10]. Recognized as one of the main factors contributing to poor asthma control, adherence to asthma treatment remains suboptimal in over 50% of cases, despite numerous studies demonstrating the effectiveness of inhaled therapy [9,11,12,13,14]. Although poor treatment adherence is associated with higher rates of morbidity, mortality, and asthma exacerbations [9,10,14,15], studies show that a significant percentage of patients do not use their medication as prescribed, either by missing doses or by using the inhaler incorrectly [4,9,13,14]. In this context, distinguishing between nonadherence to treatment and the intrinsic severity of the disease poses a major challenge in clinical practice [9,16]. Treatment adherence, asthma control, and quality of life are related but distinct aspects of asthma management. Evaluating them together may clarify how treatment-related and clinical factors are associated with specific quality-of-life domains.

According to the World Health Organization, quality of life is defined as an individual’s perception of their place in life, within the context of the cultural and value systems in which they live and in relation to their goals, expectations, and standards, representing a multidimensional concept that integrates physical health, psychological well-being, level of autonomy, social relationships, personal beliefs, and characteristics of the living environment [6,8]. To optimize the treatment of bronchial asthma, it is essential to use practical tools that comprehensively assess patients’ quality of life and are cost-effective, valid, and reliable [17]. Assessing treatment adherence is a complex process involving a range of methods, from validated self-assessment tools and drug concentration testing to electronic monitoring of inhaler use [9,15]. However, given the difficulty of applying other monitoring methods in current clinical practice due to procedural complexity or cost, validated questionnaires and self-assessment tools remain widely used [12].

Accurate assessment of treatment adherence and inhaler technique is essential in asthma management because suboptimal medication use may contribute to poor disease control and complicate the evaluation of treatment response. The Test of Adherence to Inhalers (TAI) is a validated instrument that quantifies adherence to inhaled therapy and helps identify different patterns of nonadherence [12,15,18].

In addition to adherence, asthma control and disease-specific quality of life can be assessed using validated patient-reported outcome measures. The five-item Asthma Control Test (ACT) evaluates symptoms, nocturnal awakening, rescue-medication use, activity interference, and patients’ perception of asthma control during the preceding four weeks. The 32-item Standardised Asthma Quality of Life Questionnaire (AQLQ (S)) assesses symptoms, activity limitation, emotional functioning, and environmental stimuli during the preceding two weeks. Both instruments have demonstrated satisfactory validity, reliability, and responsiveness [6,8,19,20]. Together with the TAI and routine clinical assessments, these instruments provide complementary information on adherence, asthma control, and the patient-perceived impact of the disease.

Although asthma control, inhaler adherence, lung function, and demographic characteristics have been associated with overall asthma-related quality of life [6,8,19], their adjusted relationships with individual AQLQ domains remain insufficiently characterized. Analyses based primarily on the total AQLQ score may obscure distinct associations with activity limitation, symptoms, emotional functioning, and environmental stimuli.

This study aimed to examine the adjusted associations of asthma control, inhaler adherence, lung function, and age with the different domains of asthma-related quality of life using a domain-specific multivariable approach.

## 2. Materials and Methods

### 2.1. Study Design

This was an observational, cross-sectional study conducted during routine outpatient clinical practice. All variables were collected at a single point in time during a scheduled clinical evaluation. The study was conducted at the “Dr. Victor Babes” Clinical Hospital for Infectious Diseases and Pulmonary Physiology in Timisoara, Romania, between January 2025 and December 2025. All assessments were conducted at the Pulmonology Clinic during routine follow-up visits, using standardized procedures applied to all participants. This study was conducted and reported in accordance with the Strengthening the Reporting of Observational Studies in Epidemiology (STROBE) guidelines [21].

### 2.2. Patient and Public Involvement

Patients or the public were not involved in the design, conduct, reporting, or dissemination plans of our research.

Participants were recruited consecutively if they had a pre-existing, physician-confirmed diagnosis of asthma documented in their medical records. Eligibility for the study was verified according to the diagnostic criteria of the 2025 Global Initiative for Asthma (GINA) strategy report. Inhaler device type was recorded as a descriptive treatment characteristic and categorized as either pMDI or DPI. All participants were of Caucasian ethnicity.

### 2.3. Inclusion and Exclusion Criteria

Eligible participants were adults aged ≥ 18 years with a physician-confirmed diagnosis of bronchial asthma established according to the clinical and functional criteria of the Global Initiative for Asthma (GINA). Participants were required to be receiving maintenance inhaled therapy, to understand their prescribed treatment regimen—including the medication type, dose, and administration frequency—and to demonstrate correct technique for their prescribed inhaler. Additional eligibility requirements were the ability to understand and complete the ACT, TAI, and AQLQs; a stable clinical condition without an acute asthma exacerbation or respiratory infection at the time of assessment; and provision of written informed consent.

Patients were excluded if they demonstrated incorrect inhaler technique or insufficient knowledge of their prescribed treatment regimen, defined as a combined score < 4 on physician-assessed TAI items 11 and 12. Other exclusion criteria were an acute asthma exacerbation or respiratory infection at the time of assessment and significant chronic respiratory disease other than asthma, such as chronic obstructive pulmonary disease or pulmonary fibrosis. Patients who had received antihistamines or oral or intravenous corticosteroids during the preceding 7 days were also excluded, as were those receiving maintenance oral corticosteroid therapy. Additional exclusion criteria included refusal or withdrawal of informed consent and neurological or psychiatric conditions that could affect cognitive or motor performance, including dementia, Parkinson’s disease, stroke sequelae, or uncontrolled psychiatric disorders. These conditions were excluded because they could interfere with inhaler-technique assessment or questionnaire completion.

Correct inhaler technique and adequate knowledge of the prescribed regimen were predefined eligibility requirements because the study was designed to evaluate patient-reported adherence among individuals capable of using their inhaler correctly. This criterion was applied before enrolment; consequently, individuals who did not meet these requirements did not undergo the subsequent ACT, AQLQ, spirometry, and complete adherence assessments.

### 2.4. Ethical Implications

All participants received a detailed explanation of the study objectives and procedures and provided written informed consent before enrollment. The study protocol was reviewed and approved by the Ethics Committee of the Clinical Hospital for Infectious Diseases and Pneumophthisiology “Dr. Victor Babes” Timisoara, Romania (protocol code 12195). All procedures adhered to the ethical principles outlined in the Declaration of Helsinki [22].

### 2.5. Clinical Information

Demographic and clinical information, including age, sex, body mass index, smoking status, comorbidities, type of inhaled treatment, and its frequency and dosage, was collected from electronic medical records and subsequently reconfirmed during spirometry testing and through responses to the questionnaires, after demonstrating correct inhalation technique through structured interviews at the time of presentation at the Pulmonology Clinic.

All clinical, respiratory function, and inhalation technique assessments were performed by trained pulmonologists using standardized procedures applied consistently to each case. The evaluation protocol included an initial assessment of inhalation technique, and subsequently, upon demonstration of correct inhalation technique, the following were performed: lung function testing with standardized spirometry, quantification of adherence to inhaler therapy using the Test of Adherence to Inhalers questionnaire (TAI), assessment of asthma control using the Asthma Control Test (ACT score), and assessment of patients’ quality of life using the Asthma Quality of Life Questionnaire (AQLQ).

For additional characterization of treatment intensity, the GINA treatment step at study inclusion was derived from the documented maintenance regimen according to the 2025 GINA strategy.

### 2.6. Assessment of Lung Function

Lung function was assessed using the Smart PFT UI system (Medical Equipment Europe GmbH, Hammelburg, Germany), which measures ventilatory capacity in a standardized manner. Spirometry was performed in accordance with the technical procedures and quality standards of the American Thoracic Society and the European Respiratory Society [23]. From each participant’s best acceptable maneuver, we extracted key lung function parameters: forced vital capacity (FVC, in liters and % of predicted), forced expiratory volume in the first second (FEV_1_, in liters and % of predicted), and the FEV_1_/FVC ratio. These measurements were used to assess respiratory health and to evaluate the degree of airflow limitation.

Spirometry was performed while participants continued their usual prescribed maintenance inhaled therapy, without withholding their regular medication before testing. No bronchodilator was administered as part of the study assessment, and no formal bronchodilator reversibility test was performed.

### 2.7. Assessment of Adherence to Inhaled Therapy

Adherence to inhaled therapy was assessed using the official Romanian-language version of the 12-item Test of Adherence to Inhalers (TAI). Items 1–10 are completed by the patient and assess adherence-related behaviors, whereas items 11 and 12 are completed by the clinician and assess knowledge of the prescribed regimen and inhaler technique [12,15,18]. For the primary analyses, the patient-completed 10-item TAI total score was used; scores range from 10 to 50, with higher values indicating better adherence. Adherence was classified as good at 50 points, intermediate at 46–49 points, and poor at ≤45 points [12,13,18,24,25].

Exploratory subscale analyses examined sporadic nonadherence using TAI items 1–5 and intentional nonadherence using items 6–10. The clinician-completed items 11 and 12 were used for eligibility assessment and were not included in the 10-item TAI total score.

### 2.8. Assessment of Asthma Control

Asthma control was assessed using the official Romanian-language version of the five-item Asthma Control Test (ACT), a self-administered instrument originally developed and validated for evaluating asthma control [19,26,27]. The ACT is a self-administered questionnaire consisting of 5 items that assess respiratory symptoms, nighttime awakenings due to symptoms, use of rescue medication, the impact of the disease on daily activities, and overall perception of asthma control over the past 4 weeks [8,19,26]. Each item is rated on a scale from 1 to 5, with a total score ranging from 5 to 25 points; higher scores indicate better disease control [19,26,28].

According to current clinically validated cut-off points, an ACT score ≥ 20 indicates well-controlled asthma, a score between 16 and 19 suggests partial control, and a score ≤ 15 is associated with inadequate disease control. The ACT exhibits robust psychometric properties, including good internal consistency, reproducibility, and sensitivity to clinical changes, and is significantly correlated with respiratory function parameters and clinical assessments of asthma control. Due to its ease of administration and extensive validation across diverse populations, the ACT is frequently used in both clinical practice and clinical trials to monitor asthma control in patients [29]. In the present study, the ACT score was used continuously in the statistical analysis.

### 2.9. Assessing Quality of Life in Asthma

Asthma-related quality of life was assessed using the self-administered Standardised Asthma Quality of Life Questionnaire (AQLQ (S)-SA), Romanian version for Romania (MAPI Institute version dated 20 September 2012; ID7034). The AQLQ (S)-SA is a validated 32-item instrument that evaluates patients’ experiences during the preceding two weeks and comprises four domains: symptoms (12 items), activity limitation (11 items), emotional functioning (5 items), and environmental stimuli (4 items) [8,19,30,31,32].

Each item is scored from 1 to 7, with higher scores indicating better asthma-related quality of life [6,8,17,19]. The score for each domain was calculated as the arithmetic mean of the items belonging to that domain, and the overall AQLQ (S) score was calculated as the mean of all 32 items. Consequently, the domain and overall scores range from 1 to 7.

### 2.10. Statistical Analysis

Statistical analyses were performed using MedCalc Statistical Software 23.4.0 (MedCalc Software Ltd., Ostend, Belgium). The distribution of continuous variables was assessed using the Shapiro–Wilk test. Continuous variables are presented as mean ± standard deviation or median and interquartile range (IQR), according to their distribution, while categorical variables are presented as counts and percentages.

Relationships among the principal study variables were initially explored using Spearman’s rank correlation coefficient. These analyses were considered exploratory and were used to identify potential bivariate associations. Additional exploratory analyses examined whether sporadic nonadherence, calculated as the sum of TAI items 1–5, and intentional nonadherence, calculated as the sum of TAI items 6–10, were associated with the individual domains of asthma-related quality of life.

To evaluate adjusted associations with domain-specific asthma-related quality of life, separate multivariable linear regression models were constructed for each AQLQ domain mean item score, ranging from 1 to 7, as the outcome. Each model included asthma control (ACT total score), inhaler adherence (TAI total score), age, and lung function (FEV_1_ % predicted) as predictors entered simultaneously using the enter method. Model assumptions were evaluated by assessing residual normality using the Shapiro–Wilk test and homoscedasticity using the Breusch–Pagan test. Potentially influential observations were screened using externally studentized residuals, leverage values, and Cook’s distance. Absolute externally studentized residuals > 3, leverage values > 2 (k + 1)/*n*, and Cook’s distances > 4/*n* were used as screening thresholds. Because these thresholds identify observations warranting further examination rather than prespecified criteria for exclusion, all observations were retained in the primary models. Leave-one-out sensitivity analyses were performed for every observation exceeding at least one screening threshold to determine whether the findings depended on any single case. Multicollinearity was assessed using variance inflation factors (VIFs), with values below 3 considered acceptable. Because the residuals of the symptom-domain model deviated from normality, a sensitivity analysis using heteroscedasticity-consistent HC3 standard errors was conducted for this model. Model performance was evaluated using the adjusted R^2^. Regression results are presented as unstandardized regression coefficients (B) with 95% confidence intervals and standardized regression coefficients (β). The absolute magnitudes of the standardized coefficients were used to compare the relative strength of associations among predictors measured on different scales. Given the number of domain-specific regression coefficients and exploratory correlation analyses, no formal adjustment for multiple comparisons was applied, and all reported *p*-values should be interpreted as nominal. The domain-specific coefficient-level analyses and exploratory correlations were therefore considered hypothesis-generating. For transparency, the regression results were also evaluated against a conservative Bonferroni benchmark of α = 0.05/16 = 0.0031, based on the 16 predictor coefficients examined across the four multivariable models. A two-sided nominal *p*-value < 0.05 was used to identify associations warranting further investigation.

Because TAI and ACT scores were strongly correlated, a sensitivity analysis was conducted for the emotional-functioning outcome by repeating the multivariable model without ACT. In addition, the partial correlation between TAI and emotional functioning was calculated while controlling for ACT, age, and FEV_1_ % predicted. These analyses were used to determine whether the TAI coefficient was sensitive to adjustment for asthma control and to assess a potential statistical suppression effect. The VIF for TAI was also reported specifically for the fully adjusted emotional-functioning model.

Exploratory sensitivity analyses were also performed by repeating each domain-specific model with additional adjustment for BMI, smoking status (never, former, or current smoker), and the presence of at least one recorded comorbidity. These expanded models were considered exploratory because the a priori sample-size calculation was based on the four predictors included in the primary models.

Before patient recruitment, an a priori sample-size calculation was performed for the planned multivariable linear regression analyses, which included four predictors: ACT, TAI, age, and FEV_1_ % predicted. Assuming a medium effect size (Cohen’s f^2^ = 0.15), a two-sided α level of 0.05, and 80% power for the overall *F*-test, approximately 90 participants were required. The final sample of 92 participants met this estimated requirement for the overall four-predictor regression model. However, the calculation was based on the overall model *F*-test and did not guarantee adequate statistical power to detect small effects of individual predictors.

## 3. Results

A total of 139 adults with asthma were screened for eligibility. Of these, 47 (33.8%) did not proceed to enrolment because they demonstrated incorrect inhaler technique or inadequate knowledge of their prescribed inhaler regimen, corresponding to a score < 4 on the physician-assessed TAI items 11 and 12. Because this eligibility criterion was applied before enrolment, these individuals did not undergo the subsequent ACT, AQLQ, spirometry, and complete adherence assessments. The remaining 92 participants fulfilled all eligibility criteria and were included in the analysis. The baseline demographic, clinical, functional, and treatment-related characteristics of the study population are presented in Table 1. The median age was 53.5 years (IQR 38.5–68.0), and 60 participants (65.2%) were female. The median body mass index was 26.9 kg/m^2^ (IQR 23.6–30.8). More than half of the participants had at least one comorbidity (57.6%), and most were never smokers (58.7%).

Lung function assessment showed a mean FEV_1_ of 79.4 ± 22.3% predicted and a mean FEV_1_/FVC ratio of 73.2 ± 11.9%. The median ACT score was 20.0 (IQR 16.0–22.0), while the median TAI score was 45.5 (IQR 41.0–48.0).

Most participants were receiving treatment corresponding to GINA Step 4 (59/92, 64.1%), while 23 (25.0%) were receiving Step 3 treatment and 10 (10.9%) were receiving Step 5 treatment.

GINA treatment step was derived from the maintenance regimen documented at study inclusion according to the 2025 GINA strategy. Step 3 included low-dose maintenance ICS–LABA or medium-dose ICS, Step 4 included medium- or high-dose maintenance ICS–LABA, and Step 5 included ICS–LABA–LAMA triple therapy. GINA, Global Initiative for Asthma; ICS, inhaled corticosteroid; LABA, long-acting β_2_-agonist; LAMA, long-acting muscarinic antagonist.

Bivariate associations among inhaler adherence, asthma control, lung function, age, and domain-specific asthma-related quality of life were examined using Spearman’s rank correlation and are presented in Table 2.

Asthma control showed strong positive correlations with all aspects of asthma-related quality of life, with correlation coefficients ranging from ρ = 0.388 for emotional function to ρ = 0.881 for symptoms (all *p* < 0.001). Inhaler adherence (TAI total score) had moderate correlations with activity limitation (ρ = 0.362, *p* = 0.0004), symptoms (ρ = 0.597, *p* < 0.0001), and environmental triggers (ρ = 0.359, *p* = 0.0004), while no link was found with emotional function (*p* = 0.868). Lung function (FEV_1_ % predicted) was moderately related to all domains of asthma-related quality of life, whereas age was mainly negatively associated with activity limitation and symptoms. Additional exploratory analyses demonstrated significant positive correlations between FEV_1_ (% predicted) and ACT score (ρ = 0.487, *p* < 0.0001), as well as AQLQ total score (ρ = 0.563, *p* < 0.0001), whereas no significant association was observed between FEV_1_ and TAI total score (ρ = 0.163, *p* = 0.1208).

Exploratory analyses assessing sporadic nonadherence to treatment (items 1–5 of the TAI), demonstrated significant positive correlations with the AQLQ activity limitation (ρ = 0.258, *p* = 0.0132), symptoms (ρ = 0.478, *p* < 0.0001), and environmental stimuli domains (ρ = 0.271, *p* = 0.0090), whereas no significant association was observed with the emotional function domain (ρ = −0.080, *p* = 0.4471). Intentional nonadherence (TAI items 6–10) was related to activity limitation (ρ = 0.424, *p* < 0.0001), environmental stimuli (ρ = 0.385, *p* = 0.0001), and symptom domains (ρ = 0.646, *p* < 0.0001), while no significant link was found with emotional functioning (*p* = 0.425). We observed also significant positive correlations between asthma control (ACT score) and all adherence-related measures, including TAI total score (ρ = 0.653, *p* < 0.0001), TAI 1–5 score (ρ = 0.533, *p* < 0.0001), and TAI 6–10 score (ρ = 0.681, *p* < 0.0001), indicating that better adherence behavior was associated with improved asthma control.

To examine adjusted associations with domain-specific asthma-related quality of life, separate multivariable linear regression models were constructed for each AQLQ domain (Table 3).

Residual normality was acceptable for the activity-limitation (Shapiro–Wilk W = 0.980, *p* = 0.168), emotional-functioning (W = 0.973, *p* = 0.054), and environmental-stimuli models (W = 0.991, *p* = 0.762), but not for the symptom-domain model (W = 0.953, *p* = 0.002). The Breusch–Pagan test showed no evidence of heteroscedasticity in the activity-limitation (*p* = 0.818), symptom (*p* = 0.169), emotional-functioning (*p* = 0.238), or environmental-stimuli model (*p* = 0.477). Because the same participants and predictors were included in all four models, the VIF values were identical across models: 2.280 for ACT, 1.773 for TAI, 1.284 for age, and 1.676 for FEV_1_ % predicted.

Cook’s distance exceeded the screening threshold of 4/*n* in seven observations in the activity-limitation model, five in the symptom model, four in the emotional-functioning model, and seven in the environmental-stimuli model. Seven observations exceeded the leverage threshold of 2 (k + 1)/*n* in each model. One observation had an absolute externally studentized residual > 3 in each of the activity-limitation and symptom models, whereas none exceeded this threshold in the emotional-functioning or environmental-stimuli models. Overall, 12, 9, 11, and 12 observations met at least one screening criterion in the activity-limitation, symptom, emotional-functioning, and environmental-stimuli models, respectively. Leave-one-out analyses of these observations showed that the direction and nominal statistical significance of the ACT associations in all four models, the age associations with activity limitation and symptoms, and the inverse TAI–emotional-functioning association remained unchanged. For the latter association, B ranged from −0.103 to −0.086 and nominal *p*-values ranged from 0.0015 to 0.0127. The FEV_1_–environmental-stimuli coefficient remained positive, but its nominal *p*-value ranged from 0.015 to 0.102, indicating sensitivity to individual observations. Thus, no single observation accounted for the principal ACT, age, or TAI coefficients, whereas the nominal FEV_1_–environmental-stimuli association was less stable. In the symptom-domain sensitivity analysis using HC3 robust standard errors, the results remained materially unchanged: ACT, B = 0.240 (95% robust CI 0.190–0.291; *p* < 0.001); TAI, B = 0.019 (95% robust CI −0.025–0.063; *p* = 0.390); age, B = −0.011 (95% robust CI −0.021–−0.002; *p* = 0.019); and FEV_1_ % predicted, B = 0.002 (95% robust CI −0.006–0.010; *p* = 0.672). The original linear regression estimates were therefore retained as the primary results.

Comparison of the absolute standardized coefficients showed that ACT had the largest standardized coefficient within each of the four domain-specific models: activity limitation (β = 0.643), symptoms (β = 0.791), emotional functioning (β = 0.547), and environmental stimuli (β = 0.339). Thus, the relative importance of ACT across the models was supported by standardized effect estimates rather than by comparisons of unstandardized coefficients or statistical significance. Age had the second-largest absolute standardized coefficient in the activity-limitation model (β = −0.324) and was also associated with the symptom domain (β = −0.137). FEV_1_ % predicted had the second-largest absolute standardized coefficient in the environmental-stimuli model (β = 0.235). In the emotional-functioning model, TAI had the second-largest absolute standardized coefficient (β = −0.372); however, as demonstrated by the bivariate and ACT-excluded analyses, this coefficient was sensitive to adjustment for asthma control and should not be interpreted as a robust measure of the substantive importance of adherence. All *p*-values reported for the regression coefficients are nominal and were not adjusted for multiple comparisons. When the results were considered against a conservative Bonferroni benchmark of α = 0.0031 for the 16 predictor coefficients, the ACT associations with activity limitation, symptoms, and emotional functioning, together with the association between age and activity limitation, met this threshold. The inverse TAI–emotional-functioning coefficient (*p* = 0.0038), the ACT–environmental-stimuli coefficient (*p* = 0.0107), the FEV_1_–environmental-stimuli coefficient (*p* = 0.038), and the age–symptom coefficient (*p* = 0.017) did not meet the Bonferroni benchmark. These coefficient-level findings should therefore be considered exploratory.

In the fully adjusted emotional-functioning model, TAI showed a nominal inverse association with the AQLQ emotional-functioning score (B = −0.094, 95% CI −0.158–−0.031; standardized β = −0.372; nominal *p* = 0.0038), although this result did not meet the conservative Bonferroni benchmark of α = 0.0031. Thus, after adjustment for ACT, age, and FEV_1_ % predicted, higher TAI scores were conditionally associated with lower emotional-functioning scores. TAI and ACT were strongly correlated (Spearman’s ρ = 0.653, *p* < 0.001), although the VIF for TAI in this model was 1.77. The partial correlation between TAI and emotional functioning after controlling for ACT, age, and FEV_1_ % predicted was r = −0.303 (*p* = 0.0033). By contrast, the unadjusted association was essentially null (Spearman’s ρ = −0.018, *p* = 0.868). Furthermore, when ACT was excluded while retaining age and FEV_1_ % predicted, the TAI coefficient was markedly attenuated and was no longer statistically significant (B = −0.015, 95% CI −0.067–0.037; *p* = 0.562). The change from a null marginal association to an inverse adjusted association, together with the disappearance of the association after ACT was removed, is consistent with a statistical suppression effect.

In exploratory sensitivity analyses additionally adjusted for BMI, smoking status, and the presence of at least one comorbidity, the associations between ACT and all four AQLQ domains remained present (Supplementary Table S1). The inverse association between TAI and emotional functioning persisted but was attenuated (B = −0.088, 95% CI −0.153–−0.023; nominal *p* = 0.0083). BMI and smoking status were not associated with any AQLQ domain. The presence of at least one comorbidity was associated with a lower activity-limitation score (B = −0.494, 95% CI −0.962–−0.026; nominal *p* = 0.0386). The association between age and the symptom domain was not retained in the expanded model.

Overall, the primary and sensitivity analyses identified asthma control as the most consistent correlate of the four asthma-related quality-of-life domains.

## 4. Discussion

In this outpatient cohort of adults with asthma, asthma control was consistently associated with all domains of asthma-related quality of life. The relationship between inhaler adherence and emotional functioning was model-dependent: TAI was not associated with emotional functioning in the bivariate analysis, whereas an inverse association emerged after adjustment for ACT, age, and lung function. This association disappeared when ACT was removed from the model, indicating that it may reflect statistical suppression related to the strong relationship between adherence and asthma control rather than a direct effect of adherence on emotional well-being. The findings therefore emphasize the multidimensional nature of asthma-related quality of life while also demonstrating the need for caution when interpreting conditional associations between correlated predictors.

Asthma control showed strong and consistent links with all areas of the Asthma Quality of Life Questionnaire in both correlation and multivariable analyses. This aligns with previous research indicating that poor asthma control is closely linked to a lower quality of life [6,19,26,33,34]. For instance, Juniper et al., who initially developed the Asthma Quality of Life Questionnaire, showed that worsening asthma symptoms and poorer disease management were strongly associated with limitations in daily activities and increased symptom burden [20]. Likewise, Schatz et al. reported that Asthma Control Test scores are closely related to patient-reported quality-of-life measures in routine clinical practice [35]. Our results support these findings and extend them by showing that ACT had the largest absolute standardized coefficient within each domain-specific model after accounting for adherence, lung function, and age.

The magnitude of the associations between ACT and the AQLQ symptom and activity-limitation domains should be interpreted considering the conceptual overlap between these instruments. ACT items assess symptom frequency, nocturnal awakening, rescue-medication use, activity limitation, and patients’ perceptions of asthma control, while the AQLQ symptom and activity domains assess several closely related experiences. Consequently, the strong correlation between ACT and the AQLQ symptom domain (ρ = 0.881) and the high adjusted R^2^ of the corresponding model (0.777) may partly reflect shared item content and measurement of related constructs rather than an independent predictive relationship. The regression models should therefore be interpreted as describing concurrent adjusted associations, not as demonstrating that ACT causally predicts subsequent quality-of-life outcomes.

The relationship between inhaler adherence and domain-specific quality of life was more complex than initially anticipated. In the bivariate analyses, higher TAI scores were associated with better activity limitation, symptoms, and environmental-stimuli scores, but not with emotional functioning. After adjustment for ACT, age, and FEV_1_ % predicted, the associations with activity limitation, symptoms, and environmental stimuli were attenuated and no longer statistically significant. Conversely, an inverse association with emotional functioning emerged only in the adjusted model. This pattern does not support a straightforward beneficial or adverse domain-specific effect of adherence on quality of life.

Previous studies examining the relationship between adherence and asthma-related quality of life have reported inconsistent findings [6,9]. Furthermore, Alqarni et al. found that better inhaler adherence was associated with fewer anxiety and depressive symptoms, which is opposite to the direction of the adjusted association observed in our study, although psychological symptoms in that study were assessed using HADS rather than the emotional domain of the AQLQ [36]. In our analysis, TAI was not associated with emotional functioning in the bivariate analysis, and the association was substantially attenuated and became nonsignificant when ACT was removed from the multivariable model. Given the strong correlation between TAI and ACT, this pattern is consistent with statistical suppression. Previous research has also demonstrated that adherence is embedded within complex relationships involving asthma control, perceived treatment necessity, concerns about medication, and perceived treatment intrusiveness [37]. Among patients with similar ACT scores, unmeasured differences in disease burden, treatment perceptions, or treatment burden may have contributed to the residual inverse association. However, these variables were not assessed, and this explanation remains speculative. The inverse coefficient should therefore be considered a conditional, hypothesis-generating finding rather than evidence that better adherence directly worsens emotional functioning.

The TAI was selected because it was developed specifically for inhaled therapy and permits both the quantification of adherence and the identification of different nonadherence patterns. Its psychometric properties and feasibility for routine respiratory practice have been demonstrated previously [12,15,18]. Several studies conducted in Latvia [26], Iran [38], and Bangladesh [13] demonstrated that concerns about the necessity and effectiveness of asthma treatment are key factors contributing to poor treatment adherence. At the same time, a study conducted in Saudi Arabia highlights the impact of poor treatment adherence and uncontrolled symptoms on levels of anxiety and depression among asthma patients, emphasizing the need for screening for these conditions [36].

After adjustment for asthma control, inhaler adherence, and age, FEV_1_ (% predicted) was associated only with the environmental-stimuli domain. This domain reflects patients’ responses to environmental triggers like dust, smoke, weather changes, or air pollution. Reduced lung function may heighten susceptibility to these triggers, making environmental exposures more likely to cause respiratory symptoms. Previous studies have also indicated that physiological impairment can contribute to symptoms worsening in response to environmental stimuli [39,40]. In the adjusted analysis, lung function was associated with the environmental-stimuli domain but not with the other quality-of-life domains. This pattern should be interpreted as a concurrent association rather than evidence of a domain-specific causal effect. Furthermore, although the direction of the FEV_1_–environmental-stimuli coefficient remained positive in leave-one-out analyses, its nominal statistical significance was sensitive to the exclusion of individual flagged observations. This finding should therefore be considered exploratory and requires confirmation in a larger independent sample.

In the primary models, older age was associated with lower activity-limitation and symptom-domain scores but was not associated with emotional-functioning or environmental-stimuli scores. In the expanded sensitivity analyses additionally adjusted for BMI, smoking status, and comorbidity, the age–symptom association was attenuated and no longer statistically significant, whereas the age–activity-limitation association remained nominal. Therefore, the observed age-related associations were partly model-dependent and should not be interpreted as evidence of underlying psychological or behavioral mechanisms. The baseline characteristics also warrant consideration. One-quarter of the participants were current smokers, and 57.6% had at least one recorded comorbidity, indicating a clinically heterogeneous population despite assessment during a stable disease phase. In the expanded sensitivity models, smoking status was not associated with any AQLQ domain after adjustment for the other covariates. Conversely, the presence of at least one comorbidity showed a nominal association with a lower activity-limitation score, suggesting that non-asthma health burden may contribute to perceived functional restriction.

An important methodological contribution of this study is the domain-specific analysis of asthma-related quality of life. Many previous investigations have relied on overall quality-of-life scores [6,19], which may obscure domain-specific relationships between potentially associated factors and different aspects of patient experience. By examining individual AQLQ domains, the present study identified different patterns of association for behavioral, clinical, physiological, and demographic factors across the dimensions of asthma-related quality of life. This domain-specific approach provides a more detailed understanding of patient-reported outcomes and emphasizes the need to consider multiple potentially associated factors when assessing quality of life in asthma.

One study that simultaneously assessed treatment adherence, asthma control, and quality of life is the ASCONA study conducted by Valero et al., which used the TAI, ACT, and Mini Asthma Quality of Life Questionnaire in a multicenter observational cohort of patients with moderate-to-severe asthma [41], subsequently focusing on satisfaction specifically regarding the type of inhaler [25]. The results showed that higher levels of adherence to inhaler therapy were associated with better asthma control and higher quality-of-life scores, suggesting an interdependent relationship between these clinical dimensions [41]. However, the analysis was conducted primarily at the aggregate level of scores, without detailed exploration of individual quality-of-life domains, which limits our understanding of the differentiated impact of adherence on specific components of quality of life.

From a clinical standpoint, the consistent associations between ACT and the AQLQ domains emphasize the importance of assessing both asthma control and quality of life in routine practice. However, the magnitude of the associations with the symptom and activity-limitation domains should be interpreted cautiously because these instruments assess partially overlapping aspects of the patient experience. The present findings do not establish a direct emotional benefit or adverse emotional effect of inhaler adherence. The conditional inverse association observed after ACT adjustment did not meet the conservative multiplicity benchmark and should be regarded as exploratory and hypothesis-generating, requiring confirmation in an independent longitudinal study. These observations highlight the importance of a comprehensive, patient-centered approach to asthma care that addresses both physiological control of the disease and behavioral factors related to treatment adherence. Looking ahead, given that past efforts have focused on developing a set of practical tools to improve and monitor treatment adherence based on the TAI questionnaire (TAI Toolkit) [15]. Future studies should evaluate interventions tailored to different patterns of nonadherence and examine their associations with changes in specific quality-of-life domains rather than focusing exclusively on the overall AQLQ score.

Several limitations should be acknowledged. First, the cross-sectional design precludes causal inference, and the temporal direction of the observed associations cannot be determined. Although the sample size met the estimated requirement for the overall regression models, statistical power to detect small effects of individual predictors was limited. Consequently, nonsignificant coefficients, particularly those with wide confidence intervals, should not be interpreted as definitive evidence of no association. Second, adherence was assessed using a self-reported questionnaire and may therefore have been affected by recall and social-desirability bias. Third, the exclusion of 47 of the 139 screened patients because of incorrect inhaler technique or insufficient knowledge of their prescribed regimen represents a potential source of selection bias. Although this predefined criterion was intended to evaluate patient-reported adherence among individuals capable of using their inhaler correctly, it resulted in a selected, technique-competent population. Patients with poorer inhaler-related behaviors may consequently have been underrepresented, potentially restricting the variability of TAI scores and attenuating or otherwise distorting the associations between adherence and quality-of-life outcomes. Because the excluded individuals did not undergo the complete study assessments, a full-cohort sensitivity analysis was not possible. The findings involving TAI should therefore be interpreted within the context of this selected population and should not be generalized to patients with incorrect inhaler technique or inadequate knowledge of their treatment regimen. In addition, the median ACT score of 20.0 and mean FEV_1_ of 79.4% predicted indicate that, on average, the participants had relatively well-controlled asthma and preserved lung function at the time of assessment. Because participants were evaluated during clinical stability and patients with an acute exacerbation, recent systemic corticosteroid treatment, or maintenance oral corticosteroid therapy were excluded, individuals with severe, unstable, or difficult-to-treat asthma may have been underrepresented. Previous exacerbation and hospitalization histories were not systematically collected for the main cohort and could not be included as potential confounders. Therefore, residual confounding by these and other unmeasured clinical characteristics cannot be excluded. The findings may therefore not be generalizable to patients with marked airflow limitation, frequent exacerbations, maintenance oral corticosteroid requirements, or other features of severe asthma. Nevertheless, ACT and FEV_1_ should not be interpreted as direct measures of asthma severity, which is defined partly by the treatment intensity required to achieve disease control. Although GINA treatment step was reported to characterize treatment intensity, formal asthma severity was not assessed, and severity-stratified analyses were not performed because the treatment-step subgroups were small and unevenly distributed, particularly the Step 5 group (*n* = 10). Such subgroup analyses would have had limited statistical power; larger studies should examine whether these associations differ between mild, moderate, and severe asthma. Finally, this was a single-center study conducted in a routine clinical-practice setting, which may limit the generalizability of the findings to other healthcare systems and more diverse asthma populations. Additionally, the inverse association between TAI and emotional functioning emerged only after adjustment for ACT and was absent from the bivariate and ACT-excluded analyses. Moreover, ACT and the AQLQ symptom and activity-limitation domains contain conceptually overlapping content, including symptoms, activity interference, nocturnal manifestations, and rescue-medication use. This overlap may have inflated the observed correlations and the explanatory performance of the corresponding regression models, particularly the symptom-domain model. These findings should therefore be interpreted as concurrent associations between related patient-reported constructs rather than evidence of independent causal prediction.

## 5. Conclusions

Among adults with asthma and correct inhaler technique, ACT showed the most consistent concurrent association with the AQLQ domains and had the largest absolute standardized coefficient within each domain-specific model. However, its strong associations with the symptom and activity-limitation domains should be interpreted in the context of conceptual overlap between the instruments. After adjustment, older age was associated with lower activity-limitation and symptom-domain scores. FEV_1_ % predicted showed a positive association with the environmental-stimuli domain, although its nominal statistical significance was sensitive to individual observations and this finding should therefore be considered exploratory. The inverse TAI–emotional-functioning coefficient emerged only after adjustment for asthma control, was absent from the bivariate and ACT-excluded analyses and did not meet the conservative Bonferroni benchmark. It should therefore be considered a model-dependent, hypothesis-generating observation rather than an established association. These findings indicate that patterns of association differ across individual AQLQ domains and support domain-specific assessment rather than reliance exclusively on the overall score.

## Figures and Tables

**Table 1:** Baseline demographic, clinical, functional, and treatment-related characteristics of the study population.

Variable	Median (IQR)/Mean ± SD
Age, years	53.5 (38.5–68.0)
Sex–Male, *n* (%)	32 (34.8)
Sex–Female, *n* (%)	60 (65.2)
BMI, kg/m^2^	26.9 (23.6–30.8)
Comorbidity, *n* (%)	
None	39 (42.4)
≥1 comorbidity	53 (57.6)
Smoking status, *n* (%)	
Never smoker	54 (58.7)
Former smoker	15 (16.3)
Current smoker	23 (25.0)
FVC, L	3.20 ± 1.10
FVC, % predicted	89.0 (77.0–100.0)
FEV_1_, L	2.43 ± 0.96
FEV_1_, % predicted	79.4 ± 22.3
FEV1/FVC ratio	73.2 ± 11.9
TAI total score	45.5 (41.0–48.0)
ACT total score	20.0 (16.0–22.0)
AQLQ–Activity limitation	4.65 ± 1.25
AQLQ–Symptoms	5.38 (4.13–6.21)
AQLQ–Emotional function	4.00 (3.40–4.80)
AQLQ–Environmental stimuli	4.35 ± 1.40
AQLQ–Total	4.73 (3.93–5.64)
Inhaler device–pMDI, *n* (%)	41 (44.6)
Inhaler device–DPI, *n* (%)	51 (55.4)
GINA treatment step, *n* (%)	Step 3: 23 (25.0); Step 4: 59 (64.1); Step 5: 10 (10.9)

Data are presented as median (interquartile range) or mean ± standard deviation, as appropriate. Abbreviations: ACT, Asthma Control Test; AQLQ, Asthma Quality of Life Questionnaire; BMI, body mass index; DPI, dry powder inhaler; FEV_1_, forced expiratory volume in 1 s; FVC, forced vital capacity; pMDI, pressurized metered-dose inhaler; TAI, Test of Adherence to Inhalers; GINA, Global Initiative for Asthma.

**Table 2:** Spearman correlations between adherence, asthma control, lung function, and domain-specific asthma-related quality of life.

Predictor	AQLQ–Activity Limitation	AQLQ–Symptoms	AQLQ–Emotional Function	AQLQ–Environmental Stimuli
ACT total score	ρ = 0.726, *p* < 0.0001	ρ = 0.881, *p* < 0.0001	ρ = 0.388, *p* = 0.0001	ρ = 0.529, *p* < 0.0001
TAI total score	ρ = 0.362, *p* = 0.0004	ρ = 0.597, *p* < 0.0001	ρ = −0.018, *p* = 0.868	ρ = 0.359, *p* = 0.0004
FEV_1_ (% predicted)	ρ = 0.563, *p* < 0.0001	ρ = 0.479, *p* < 0.0001	ρ = 0.338, *p* = 0.0010	ρ = 0.486, *p* < 0.0001
Age (years)	ρ = −0.538, *p* < 0.0001	ρ = −0.332, *p* = 0.0012	ρ = −0.205, *p* = 0.0501	ρ = −0.313, *p* = 0.0024

Spearman’s rank correlation coefficients (ρ) are shown. The analyses were exploratory, and the reported *p*-values are nominal and were not adjusted for multiple comparisons. Abbreviations: ACT, Asthma Control Test; AQLQ, Asthma Quality of Life Questionnaire; FEV_1_, forced expiratory volume in 1 s; TAI, Test of Adherence to Inhalers.

**Table 3:** Multivariable linear regression models examining factors associated with domain-specific asthma-related quality of life.

AQLQ Domain (Adjusted R^2^)	Predictor	B (95% CI)	Standardized β	*p*-Value
Activity limitation (0.662)	ACT total score	0.174 (0.125–0.224)	0.643	<0.001
	TAI total score	−0.012 (−0.053–0.029)	−0.048	0.556
	Age, years	−0.024 (−0.034–−0.014)	−0.324	<0.001
	FEV_1_, % predicted	0.006 (−0.003–0.015)	0.105	0.185
Symptoms (0.777)	ACT total score	0.240 (0.195–0.285)	0.791	<0.001
	TAI total score	0.019 (−0.018–0.057)	0.067	0.310
	Age, years	−0.011 (−0.020–−0.002)	−0.137	0.017
	FEV_1_, % predicted	0.002 (−0.006–0.010)	0.027	0.670
Emotional functioning (0.195)	ACT total score	0.148 (0.072–0.224)	0.547	0.0002
	TAI total score	−0.094 (−0.158–−0.031)	−0.372	0.0038
	Age, years	−0.001 (−0.017–0.014)	−0.015	0.890
	FEV_1_, % predicted	0.005 (−0.009–0.018)	0.088	0.472
Environmental stimuli (0.325)	ACT total score	0.103 (0.025–0.181)	0.339	0.0107
	TAI total score	0.026 (−0.039–0.090)	0.090	0.437
	Age, years	−0.009 (−0.025–0.007)	−0.112	0.255
	FEV_1_, % predicted	0.015 (0.001–0.029)	0.235	0.038

Each AQLQ domain score was calculated as the mean of its constituent item scores and therefore ranged from 1 to 7, irrespective of the number of items in the domain; higher scores indicate better quality of life. B represents the unstandardized regression coefficient, whereas β represents the standardized regression coefficient. Standardized β coefficients were used to compare the relative magnitude of associations among predictors and across models; unstandardized B coefficients were not used for this purpose. Each model included ACT score, TAI score, age, and FEV_1_ % predicted, entered simultaneously using the enter method. Model fit is reported as adjusted R^2^ beside each domain name. The reported *p*-values are nominal and were not adjusted for multiple comparisons. A conservative Bonferroni benchmark across the 16 predictor coefficients would correspond to α = 0.0031. All 92 participants were retained in the primary models; the influence of observations meeting the diagnostic screening thresholds was examined using leave-one-out sensitivity analyses. Abbreviations: ACT, Asthma Control Test; AQLQ, Asthma Quality of Life Questionnaire; CI, confidence interval; FEV_1_, forced expiratory volume in 1 s; TAI, Test of Adherence to Inhalers.

## Data Availability

The data supporting the findings of this study are available within the article. Additional extracted data are available from the corresponding author upon reasonable request.

## Supplementary Materials

The following supporting information can be downloaded at: https://www.mdpi.com/article/10.3390/healthcare14162626/s1, Table S1: Exploratory sensitivity analyses additionally adjusted for BMI, smoking status, and comorbidity.

## Abbreviations

The following abbreviations are used in this manuscript:

ACT	Asthma Control Test
AQLQ	Asthma Quality of Life Questionnaire
ATS	American Thoracic Society
BMI	Body Mass Index
CI	Confidence Interval
COPD	Chronic Obstructive Pulmonary Disease
DPI	Dry Powder Inhaler
ERS	European Respiratory Society
FEV_1_	Forced Expiratory Volume in the First Second
FVC	Forced Vital Capacity
GINA	Global Initiative for Asthma
pMDI	Pressurized Metered-Dose Inhaler
SD	Standard Deviation
TAI	Test of Adherence to Inhalers
VIF	Variance Inflation Factor
