# Supplementary material for: Factors Associated with Domain-Specific Asthma-Related Quality of Life in Adults with Asthma
Source: Healthcare (Basel). 2026 Aug 19;14(16):2626. doi: 10.3390/healthcare14162626 (PMC13512896; doi:10.3390/healthcare14162626)
Supplement: Supplementary file 1 [file healthcare-14-02626-s001.zip › healthcare-4436737-supplementary.pdf]

Supplementary Table S1. Exploratory sensitivity analyses additionally adjusted for BMI, smoking status, and comorbidity

AQLQ domain (adjusted R^2)	Predictor	B (95% CI)	Standardized β	Nominal p-value
**Activity limitation (0.670)**	ACT total score	0.164 (0.114 to 0.215)	0.605	<0.001
	TAI total score	−0.011 (−0.052 to 0.031)	−0.043	0.601
	Age	−0.015 (−0.028 to −0.001)	−0.203	0.030
	FEV_1 % predicted	0.003 (−0.006 to 0.012)	0.059	0.469
	BMI	−0.006 (−0.034 to 0.021)	−0.029	0.652
	Current smoker	−0.219 (−0.698 to 0.261)	−0.065	0.367
	Former smoker	−0.121 (−0.501 to 0.260)	−0.042	0.530
	≥1 comorbidity	−0.494 (−0.962 to −0.026)	−0.196	0.039
**Symptoms (0.777)**	ACT total score	0.236 (0.183 to 0.290)	0.778	<0.001
	TAI total score	0.023 (−0.024 to 0.070)	0.080	0.339
	Age	−0.004 (−0.018 to 0.011)	−0.043	0.625
	FEV_1 % predicted	0.000 (−0.009 to 0.009)	0.002	0.978
	BMI	−0.003 (−0.030 to 0.024)	−0.012	0.837

	Current smoker	0.021 (−0.490 to 0.532)	0.006	0.936
	Former smoker	−0.138 (−0.508 to 0.232)	−0.043	0.461
	≥1 comorbidity	−0.357 (−0.785 to 0.071)	−0.126	0.101
**Emotional functioning (0.187)**	ACT total score	0.145 (0.066 to 0.224)	0.538	<0.001
	TAI total score	−0.088 (−0.153 to −0.023)	−0.348	0.008
	Age	0.007 (−0.014 to 0.028)	0.094	0.517
	FEV_1 % predicted	0.003 (−0.011 to 0.017)	0.052	0.683
	BMI	0.013 (−0.030 to 0.056)	0.060	0.558
	Current smoker	0.119 (−0.631 to 0.869)	0.035	0.753
	Former smoker	−0.382 (−0.977 to 0.213)	−0.133	0.205
	≥1 comorbidity	−0.339 (−1.072 to 0.393)	−0.135	0.360
**Environmental stimuli (0.310)**	ACT total score	0.107 (0.026 to 0.189)	0.354	0.011
	TAI total score	0.032 (−0.035 to 0.099)	0.112	0.346
	Age	−0.002 (−0.023 to 0.020)	−0.021	0.875
	FEV_1 % predicted	0.015 (0.001 to 0.030)	0.243	0.041

	BMI	−0.016 (−0.061 to 0.028)	−0.067	0.474
	Current smoker	0.360 (−0.415 to 1.136)	0.096	0.358
	Former smoker	0.177 (−0.437 to 0.792)	0.055	0.568
	≥1 comorbidity	−0.248 (−1.005 to 0.509)	−0.088	0.517

Values are unstandardized regression coefficients (B) with 95% confidence intervals, standardized coefficients (β), and nominal p-values. Each model included ACT total score, TAI total score, age, FEV_1 % predicted, BMI, smoking status, and the presence of at least one comorbidity. Never smokers were the reference category for smoking status, and absence of comorbidity was the reference category for comorbidity. Because residuals from the symptom-domain model deviated from normality, its confidence intervals and p-values were calculated using HC3 robust standard errors. No adjustment for multiple comparisons was applied. AQLQ, Asthma Quality of Life Questionnaire; ACT, Asthma Control Test; BMI, body mass index; CI, confidence interval; FEV_1, forced expiratory volume in one second; TAI, Test of Adherence to Inhalers.
